# Preference differences of different styles of oil paintings in various interior environments based on the PAD emotional state model and EEG

**DOI:** 10.3389/fpsyg.2025.1713079

**Published:** 2026-01-30

**Authors:** Donghai Huang, Chang Liu, Caiping Lian, Huajie Shen, Xinzhen Zhuo, Caixia Bai, Tong Tang, Rongfeng Ding

**Affiliations:** 1 School of Design, Fujian University of Technology, Fuzhou, Fujian, China; 2 Intangible Cultural Heritage Arts and Crafts Research Center, Fujian University of Technology, Fuzhou, Fujian, China; 3 Fuzhou Research Center for Public Service Advertising, Fujian University of Technology, Fuzhou, Fujian, China; 4 School of Art and Design, Qilu University of Technology (Shandong Academy of Sciences), Jinan, China

**Keywords:** aesthetic preference, decorative oil painting, EEG technology, interior environment, PAD emotion scales

## Abstract

**Introduction:**

Decorative oil paintings are an integral component of interior environments, influencing not only spatial aesthetics but also occupants’ emotional experiences and psychological perceptions. However, limited research has systematically examined the emotional interaction between different oil painting styles and interior design styles, particularly through the integration of subjective evaluations and objective physiological measures.

**Methods:**

This study investigated the emotional effects and preference characteristics of oil painting styles across various interior environments using a combined approach of the Pleasure–Arousal–Dominance (PAD) emotional scale and electroencephalography (EEG). Eight representative oil painting styles and six common interior design styles were selected. Participants’ subjective emotional responses were assessed using the PAD scale, while neurophysiological activity was recorded via EEG. Emotional preferences and neural responses were analyzed to explore the relationships among painting styles, interior styles, and emotional perception.

**Results:**

The results indicated that Impressionistic, Post-Impressionistic, and Romanticism oil paintings were generally more preferred in modern interior environments, whereas Contemporary art was less favored and occasionally elicited negative emotional responses. EEG findings were largely consistent with PAD measurements: Higher preferences for Impressionistic, Post-Impressionistic, and Romanticism paintings were associated with increased positive-going amplitudes in the left frontal regions (Fp1 and F3), while Contemporary art elicited stronger negative-going amplitudes in the right frontal regions (Fp2 and F4). Additionally, prefrontal amplitude differences suggested variations in perceptual or attentional processing demands across interior styles. American- and Nordic-style interiors enhanced emotional pleasure, whereas Pastoral-style interiors were associated with reduced cognitive engagement. Significant preference differences were also observed across age and sex groups, with older participants favoring culturally rich styles such as the New Chinese style, and younger participants preferring visually impactful styles such as Romanticism and Impressionism.

**Discussion:**

Overall, EEG patterns exhibited qualitative consistency with PAD emotional evaluations, supporting the valence hypothesis. The findings elucidate the mechanisms by which decorative oil painting styles and interior environments jointly influence emotional experiences. This study provides scientific evidence for interior design optimization, art curation, and environmental psychology research, offering practical references for enhancing visual experience and emotional congruence in interior spaces.

## 1 Introduction

Art and interior design have a long, intertwined history, both shaping human perception and experience within architectural spaces. Among various artistic forms, oil paintings are a popular choice for interior decoration due to their distinctive aesthetic value, emotional resonance, and cultural significance. However, individual preferences for oil painting styles, particularly the interior design style, are not formed in isolation but are significantly influenced by the surrounding environmental context. An in-depth understanding of how various painting styles evoke individual emotional responses and decorative preferences under various interior design styles is of theoretical and practical significance. It enhances spatial aesthetics, improves environmental comfort, and increases user satisfaction (Mehrabian and Russell, 1974; Nasar, 1994).

Previous studies on art and interior design preferences often relied on subjective self-report measures, which were prone to cognitive bias and linguistic limitations (Küller et al., 2006). The integration of psychological emotion models and neurophysiological techniques has opened new paths for exploring art appreciation and environmental psychology in recent years (Xiong, 2024; Zhang and Zhou, 2021). Among these models, the Pleasure–Arousal–Dominance (PAD) emotion scales proposed by Mehrabian and Russell (1974) have been widely applied in environmental psychology, product design, and user experience research. The PAD emotion scales quantify emotional responses through three dimensions: pleasure (positive or negative feelings), arousal (activation or relaxation), and dominance (sense of control or submission). These scales help reveal the mechanisms of emotional experiences triggered by visual stimuli (Mehrabian and Russell, 1974; Russell, 1980).

Meanwhile, electroencephalography (EEG) is a vital neurophysiological tool that allows real-time monitoring of brain activity. It reveals the neural mechanisms involved in aesthetic experience and environmental perception (Rui and Gu, 2021). EEG technology, with its high temporal resolution, can capture immediate neural responses of individuals to artistic works and spatial environments. Existing studies have demonstrated a close association of specific EEG frequency bands (*α*, *β*, and *θ* waves) with emotional processing, aesthetic experience, and cognitive engagement (Klimesch, 1999; Capotosto et al., 2009). Meanwhile, the stimulation-specific neural responses of EEG signals are often analyzed using ERPs. Emotional visual stimuli typically elicit ERP components such as (second positive peak), N2 (second negative peak), P300 (third positive peak), and the late positive potential (LPP).

Despite a recent increase in the number of investigations on emotional and neurophysiological responses triggered by visual stimuli, studies exploring the interactive effects of various oil painting styles and interior design styles on individual preferences remain limited (Vartanian et al., 2013). Existing studies often analyze oil painting and interior design separately, overlooking how their interaction influences emotional experience and preference formation. Moreover, systematic explorations into the neurophysiological mechanisms underlying such preferences are limited (Leder et al., 2004). The combination of PAD emotion scales and EEG provides a novel research perspective for connecting subjective emotional self-reports with objective neurophysiological data, thus enabling a comprehensive understanding of individual artistic preferences in specific contexts.

Therefore, this study aimed to use the PAD emotional state model and EEG to investigate emotional and neurophysiological responses of participants to different oil painting styles under various interior design styles. The major objectives were as follows: (1) to examine how oil painting and interior design styles jointly influence individual emotional responses; (2) to identify differences in EEG patterns associated with different emotional and aesthetic experiences; and (3) to provide optimization strategies and a scientific basis for the practical application of art and design. This study combined psychological emotion modeling with neurophysiological measurement to deepen interdisciplinary research in art psychology and environmental psychology, offering scientific guidance for interior designers, architects, and art curators and promoting the deep integration and innovative practice of art and environmental design.

## 2 Samples and methods

### 2.1 Sample image collection

**Figure 1:**
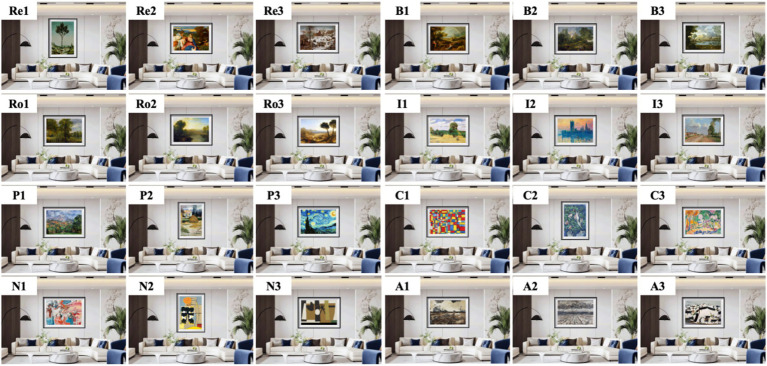
Eight styles of oil paintings in modern interior environments. Re, Renaissance; B, Baroque; Ro, Romanticism; I, Impressionistic; P, Post-Impressionistic; C, Contemporary art; M, Modernism; A, Abstractionism.

**Figure 2:**
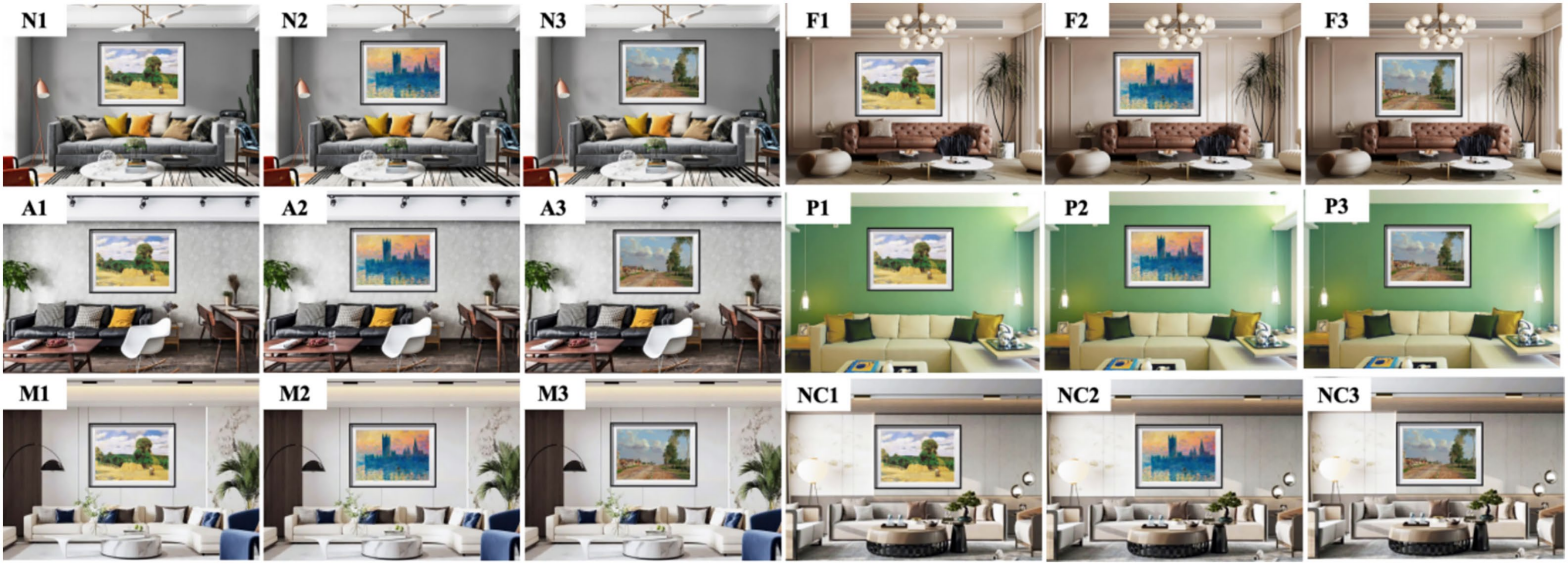
Impressionistic oil paintings displayed within six styles of interior design (N, Nordic; F, French; A, American; P, Pastoralism; M, Modern; NC, New Chinese).

We collected 42 sample images and divided them into 2 groups. The first group comprised scene images of eight styles of decorative oil paintings placed within modern-style (the most common) interior environments, including Renaissance, Baroque, Romanticism, Impressionistic, Post-Impressionistic, Contemporary art, Modernism, and Abstractionism. Three images were selected for each style. All sample images were processed using Photoshop to ensure uniform dimensions (2,958 × 1,837 pixels), with consistent brightness and clarity, as shown in Figure 1. The second group featured Impressionistic oil paintings within six common interior design styles: Nordic, French, American, Pastoralism, Modern, and New Chinese. The size and image processing levels were consistent with those in the first group, as shown in Figure 2. Three images were selected for each style, and the number of stimuli per style follows previous neuroaesthetic studies using controlled visual stimuli to reduce extraneous variability (Vartanian and Goel, 2004).

### 2.2 PAD emotional measurement experiment

#### 2.2.1 PAD emotional state model

The PAD emotional state model aimed to quantify and present the three-dimensional emotional characteristics of users in response to stimuli (Li et al., 2023). The model accurately evaluated emotional states through three basic dimensions: Pleasure (P), Arousal (A), and Dominance (D). Specifically, P mainly represented the positive and negative emotional states of an individual’s emotions. A represented an individual’s neurophysiological activation level, reflecting the degree of feeling happy, active, and stimulated in a particular situation. For example, “interest” was a high-arousal state with a positive A value, whereas “boredom” was a low-arousal state with a negative A value. D represented the degree of an individual’s control over external situations and others (Jang and Lee, 2019). Basesd on these three dimensions, emotional states were classified into eight categories, as shown in Table 1.

**Table 1:** Eight types of emotions of P, A, and D.

No.	Type of emotion		No.	Type of emotion	
1	+P + A + D	Joyful	5	−P−A−D	Boring
2	+P + A−D	Dependent	6	−P−A + D	Contempt
3	+P−A + D	Relaxed	7	−P + A−D	Anxiety
4	+P−A−D	Mild	8	−P + A + D	Disgust

**Figure 3:**
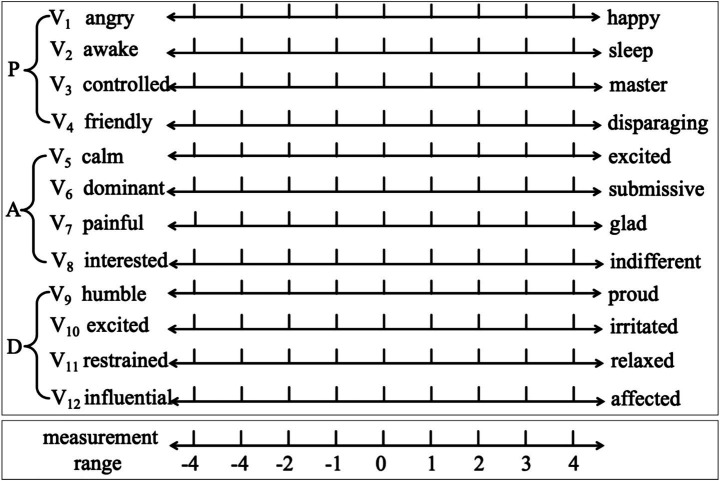
Standardized Chinese version of PAD emotion scales.

The Institute of Psychology of the Chinese Academy of Sciences developed a standardized Chinese version of the PAD model to align with Chinese emotional characteristics. This scale was a multidimensional emotion measurement tool based on the multidimensional emotion space model. The scale used a 9-point rating mechanism with positive and negative poles. Each of the three dimensions (P, A, and D) included four groups of semantically opposite adjectives to describe human emotions, as shown in Figure 3. The actual PAD measurement values were calculated using Equations 1–3. Because the PAD analysis in this study involved descriptive comparisons of mean emotional ratings across conditions, effect sizes were reported following APA recommendations for non-inferential research. Standard deviations were not required in this exploratory paradigm; therefore, effect sizes were computed using a standardized mean difference (SMD) metric suitable for descriptive data. The SMD was defined as Equation 4, which formulation provides a normalized estimate of the magnitude of difference between two emotional responses without requiring variance estimates, which is appropriate for exploratory affective and neuroaesthetic studies.


P=(V1–V4+V7–V10)/4 (1)

A=(–V2+V5–V8+V11)/4 (2)

D=(V3–V6+V9–V12)/4 (3)

Where P, A, and D represent the actual measured values of color emotion. V, values.


$$\mathrm{SMD}=\frac{M_{1}+M_{2}}{|M_{1}|+|M_{2}|}$$ (4)

Where M_1_ and M_2_ represent the mean PAD values of the two conditions being compared.

Moreover, PAD directly reflects positive and negative emotional states of individuals, with each emotional characteristic corresponding to a set of PAD normative values (Wu et al., 2024). Therefore, in this study, the eight normative values of PAD emotional characteristics listed in Table 2 were used to describe the positive and negative emotional characteristics of evaluated oil paintings in interior spaces. This enabled the objective measurement of specific emotional characteristics of participants corresponding to the actual PAD measurement structure and clarified the emotional experience orientation. The emotional preferences of participants for oil paintings were further identified using the Euclidean distance to calculate the proximity between actual measured emotion values of participants and PAD normative values (Wang et al., 2023). The minimum proximity value of the eight emotional characteristics represented the emotional preference of participants for different painting styles in various interior environments. The proximity value was calculated using Equation 5. Here, emotional preference refers to lower PAD-norm distance (Equation 5).


$$\mathrm{Li}=\sqrt{{\left(P-P_{N}\right)}^{2}+{\left(A-A_{N}\right)}^{2}+{\left(D-D_{N}\right)}^{2}}\;(i\in z)$$ (5)

Where z is a positive integer.

**Table 2:** Normative values of PAD emotional characteristics.

No.	Type of emotion	PN	AN	DN
1	Joyful	2.77	1.21	1.42
2	Relaxed	2.19	−0.66	1.05
3	Surprise	1.72	1.71	0.22
4	Dependent	0.39	−0.81	−1.48
5	Boring	−0.53	−1.25	−0.84
6	Fear	−0.95	0.32	−0.63
7	Contempt	−1.58	0.32	1.02
8	Digust	−1.8	0.4	0.67

PN, AN, and DN represent the PAD normative values.

#### 2.2.2 Emotion testing

This study used decorative oil paintings, displayed in Figures 1 and 2, as the subjects of emotion testing. The PAD emotion scales (Figure 3) were used. A total of 35 students majoring in design (20 male and 15 female students; aged 18–25 years) and 10 professionals (aged 33–56 years) specializing in environmental design participated in the experiment. Participants’ handedness, neurological and visual health, and art-training backgrounds were self-reported as normal. All were right-handed and had no history of neurological disorders. Each participant scored the 42 paintings based on the 12-item PAD emotion scale, with scores ranging from −4 to 4, as shown in Figure 3. The actual values for each emotional dimension were calculated according to the corresponding dimensions of adjective pairs using Equations 1–3. The emotional classification was performed by combining the positive and negative values of the three dimensions (Table 1). Subsequently, the emotional preference for each sample image was evaluated using the PAD normative values in Table 2 and Equation 4.

All data were analyzed using Excel and SPSS software.

### 2.3 EEG experiment

#### 2.3.1 Participants

The volunteers for the EEG test were the 35 design major students from the emotion testing, who primarily specialized in environmental design. The participants were required to take adequate rest prior to the experiment, avoid intense physical or mental labor, refrain from medication or alcohol, and maintain full alertness throughout the experiment. The study was approved by the Ethics Committee of Fujian University of Technology. All participants signed an informed consent form prior to participation, and small gifts were offered to them as compensation after the experiment.

#### 2.3.2 EEG equipment and recording parameters

The frontal lobe is primarily associated with thinking, emotion, planning, and needs. Therefore, the frontal alpha asymmetry (FAA) can be used to compare emotional differences in response to various stimuli. The left frontal region is primarily involved in processing positive emotions, whereas the right frontal region is associated with negative emotions (Briesemeister et al., 2013). This is consistent with the “valence hypothesis”: left hemisphere activity increases during positive emotional states, whereas right hemisphere activity increases during negative emotional states. Therefore, in this study, the frontal lobe was selected as the object, recording EEG data from channels FP1 (left frontal pole), FP2 (right frontal pole), F3 (left frontal), F4 (right frontal), FZ (frontal midline), CZ (central midline), and PZ (parietal midline). The 7-channel montage was focused on frontal and midline regions, which are the primary scalp locations associated with emotional valence and cognitive appraisal (Coan and Allen, 2004). Although this does not provide full scalp coverage, such reduced-channel systems have been widely used in affective computing and neuroaesthetic studies. Future research may employ high-density EEG to improve spatial resolution.

The study was conducted in a human factors engineering laboratory with a comfortable indoor temperature and no noise interference. An Australian Okti EEG equipment was used, including an EEG amplifier, electrode cap (64 electrodes), conductive gel, stimulus presentation software E-Prime, and data acquisition and analysis software Curry 9.

#### 2.3.3 E-prime procedure

Eight sample images of eight different oil painting styles (Re1, B1, Ro1, I1, P1, C1, M1, and A1; Figure 1) and six sample images of Impressionist paintings in six different interior styles (N1, F1, A1, P1, M1, and NC1; Figure 2) were selected. All these images were processed using Photoshop to maintain uniform size and eliminate the influence of irrelevant factors such as color. The experiment was conducted using E-Prime software. The participants were instructed to perceive the oil painting as a whole. Each image was randomly presented at the center of the computer screen. The participants responded based on their preference: pressing “1” for “like,” “2” for “neutral,” and “3” for “dislike.” Each image was randomly presented 40 times. The procedure continued until all 14 images were presented, and EEG data recording was terminated.

#### 2.3.4 Experimental procedure

Before the experiment, the participants were informed about the purpose, procedure, and precautions of the experiment to alleviate anxiety. The electrode cap was worn from front to back, and the electrodes were placed. An appropriate conductive gel was applied to ensure that the impedance at all leads was reduced to below 5 Hz. The participants adopted a comfortable sitting posture at approximately 50 cm from the screen and refrained from any movement during the experiment. The EEG data were recorded during the experiment using Curry 9. EEG preprocessing originally included band-pass filtering (0 ~ 30 Hz, low pass), 50 Hz notch filtering, segmentation (−200 to 800 ms), baseline correction (Constant), and re-referencing to averaged mastoids of both sides. ICA was adopted to remove ocular artifacts, of which threshold was 0 to 200 Hz. When removing the BadBlok, the threshold is selected as −100 to 100 Hz, and automatic inspection is carried out.

## 3 Results and discussion

### 3.1 PAD preference analysis

#### 3.1.1 Preference differences of eight common styles of oil paintings in modern interior environments

PAD testing across eight common oil painting styles revealed distinct preference tendencies by age and sex (Tables 3, 4). Among younger participants, Impressionistic paintings produced the highest pleasure rating (P ≈ 0.80), followed by Post-Impressionistic, Modernism, and Abstractionism, which all generated generally positive emotional responses. In contrast, Contemporary art elicited negative pleasure (P ≈ −0.25), indicating low engagement in this group. Impressionistic paintings elicited substantially higher Pleasure ratings than Contemporary art among younger participants, with a large effect size (SMD = 1.00).

**Table 3:** PAD emotion testing results and emotion categories for eight oil painting styles by age and sex.

oil painting style	Yong				Yong-man				Yong-women				elderly			
	Py	Ay	Dy	emotions type	Pm	Am	Dm	emotions type	Pw	Aw	Dw	emotions type	Pe	Ae	De	emotions type
Renaissance	0.1963	0.0786	-0.0333	Dependent	0.1667	0.0542	-0.0167	Dependent	0.0980	0.1111	-0.0556	Dependent	0.2833	-0.1167	-0.0167	Mild
Baroque	0.2333	-0.0762	0.0595	Relaxed	0.1500	-0.0875	0.0958	Relaxed	0.1432	-0.0611	0.0111	Relaxed	0.5083	-0.2583	-0.1083	Mild
Romanticism	0.4429	0.0690	-0.1881	Dependent	0.1125	0.0792	-0.1042	Dependent	0.8833	0.0556	-0.3000	Dependent	0.5583	0.0167	-0.0833	Dependent
Impressionistic	0.8000	0.2071	-0.0571	Dependent	0.4958	0.1292	-0.2167	Dependent	1.2056	0.3111	0.1556	Joyful	1.3083	0.5750	0.1250	Joyful
Post-impressionistic	0.2743	0.0619	0.0738	Joyful	-0.0583	-0.0125	0.0250	Contempt	0.5778	0.1611	0.1389	Joyful	0.9583	0.6750	0.0583	Joyful
Contemporary art	-0.2500	-0.1833	-0.0143	Boring	-0.1542	-0.0958	-0.0792	Boring	-0.3667	-0.3000	-0.0722	Boring	0.3667	-0.0750	-0.0750	Mild
modernism	0.0524	0.2976	0.4810	Joyful	0.0125	0.1667	0.1875	Joyful	0.1056	0.4722	0.8722	Joyful	0.8250	0.4250	0.1750	Joyful
abstractionism	0.1071	0.2048	0.1690	Joyful	-0.1333	0.1875	0.0417	Digust	0.4278	0.2278	0.3389	Joyful	0.5667	0.4417	0.2250	Joyful

**Table 4:** Minimum PAD emotion proximity values for eight oil painting styles by age and sex.

Volunteer	oil painting style	Positive emotion				Negative emotion			
		Joyful	Relaxed	Surprise	Dependent	Boring	Fear	Contempt	Digust
Yong	Renaissance	######	######	2.2467	1.7088	1.7156	1.3146	2.0792	2.1408
	Baroque	######	######	2.3295	1.7126	1.6642	1.4257	2.0899	2.1757
	Romanticism	######	######	2.1190	**1.5635**	1.7639	1.4827	2.3695	2.4241
	Impressionistic	**#####**	**#####**	**######**	1.7964	2.1225	1.8448	2.6148	2.7066
	Post-impressionistic	######	######	2.1972	1.7855	1.7897	1.4356	2.0977	2.1846
	Contemporary art	######	######	2.7424	1.7177	**######**	**######**	1.7584	**1.7919**
	modernism	######	######	2.2009	2.2773	2.1164	1.4965	**######**	1.8648
	abstractionism	######	######	2.2067	1.9568	1.8816	1.3302	1.8931	1.9815
Yong-man	Renaissance	######	######	2.2827	1.7141	1.6924	1.3015	2.0485	2.1116
	Baroque	######	######	2.3898	1.7501	1.6400	1.3795	2.0033	2.0904
	Romanticism	######	######	2.3127	1.6615	1.6495	1.2097	2.0460	2.0880
	Impressionistic	**#####**	**#####**	**######**	**1.5777**	1.8284	1.5158	2.4238	2.4760
	Post-impressionistic	######	######	2.4834	1.7613	1.5818	1.1553	1.8483	1.9025
	Contemporary art	######	######	2.6197	1.6639	**######**	**######**	1.8477	1.8751
	modernism	######	######	2.3018	1.9690	1.8322	1.2721	1.8035	1.8901
	abstractionism	######	######	2.4051	1.8932	1.7324	1.0657	**######**	**1.7938**
Yong-woman	Renaissance	######	######	2.2942	1.7213	1.6919	1.2132	2.0040	2.0524
	Baroque	######	######	2.3805	1.6868	1.6097	1.3234	2.0329	2.1030
	Romanticism	######	######	1.9255	**1.5443**	1.9984	1.8815	2.8072	2.8740
	Impressionistic	**#####**	**#####**	**######**	2.1441	2.5378	2.2943	2.9166	3.0506
	Post-impressionistic	######	######	1.9262	1.8971	2.0437	1.7177	2.3362	2.4481
	Contemporary art	######	######	2.9120	1.6776	**######**	**######**	1.7463	**1.7594**
	modernism	######	######	2.1363	2.6941	2.5103	1.8423	**######**	1.9176
	abstractionism	######	######	1.9700	2.0945	2.1192	1.6869	2.1222	2.2588
Elderly	Renaissance	######	######	2.3360	1.6228	1.6198	1.4450	**######**	**2.2536**
	Baroque	######	######	2.3346	**1.4832**	**######**	1.6533	2.4431	2.5234
	Romanticism	######	######	2.0758	1.6317	1.8334	1.6328	2.4252	2.5052
	Impressionistic	**#####**	######	**######**	2.3103	2.7643	2.3948	3.0346	3.1606
	Post-impressionistic	######	**#####**	1.2952	2.2124	2.5938	2.0595	2.7375	2.8387
	Contemporary art	######	######	2.2594	1.5858	1.6643	**######**	2.2682	2.3399
	modernism	######	######	1.5666	2.1103	2.3816	1.9518	2.5513	2.6714
	abstractionism	######	######	1.7143	2.1225	2.2801	1.7453	2.2924	2.4085

The “#” symbols in the table representspecific numerical values that are not displayed.The bold values indicate the smallest PAD emotion proximity value among these several types of samples style.

For the elderly group, all eight painting styles received positive pleasure scores, with Impressionistic paintings again achieving the highest rating (P ≈ 1.31). Elderly participants responded favorably to Post-Impressionistic, Romanticism, Modernism, and Abstractionism, while Renaissance, Baroque, and Contemporary art produced more neutral emotional evaluations. This age pattern is consistent with emotion-regulation theory (Gross, 2002), which suggests that older adults selectively favor experiences that support positive affect. Additionally, the difference between younger and older adults for Impressionistic and Romanticism paintings was small (SMD = 0.24; 0.12), indicating broadly consistent preference across age groups.

Arousal differences further distinguished the two age groups. Younger participants exhibited higher arousal sensitivity, consistent with developmental theories emphasizing exploration and heightened emotional experience in youth (Erikson, 1968). For instance, younger viewers reported higher arousal in Romanticism (A ≈ 0.07 vs. 0.02) and less negative arousal in Baroque (A ≈ −0.08 vs. –0.26), suggesting greater responsiveness to visually dynamic or high-contrast works. Dominance values showed minimal age differences in structurally formal styles such as Renaissance and Baroque (D ≈ −0.03 vs. –0.02), indicating comparable perceptions of control across groups.

Sex-related analyses revealed that female participants generally exhibited higher pleasure and arousal ratings, particularly in Impressionistic and Romanticism styles (e.g., Romanticism P-female ≈ 1.21 vs. P-male ≈ 0.50) with a medium effect size (SMD = 0.42), reflecting stronger emotional sensitivity (Baron-Cohen, 2003; Hanna et al., 2011). Female viewers tended to respond more to emotional tone, whereas male participants focused more on structural or technical qualities, as reflected in slightly higher dominance ratings in stylistically formal categories such as Baroque.

Overall, these findings align with prior research suggesting that aesthetic experience is shaped by age-related emotional regulation, developmental sensitivities, and sociocultural patterns of art appreciation (Chatterjee et al., 2010). Despite demographic differences, Impressionistic paintings consistently evoked the strongest positive responses, while Contemporary art generated the least interest across all groups.

#### 3.1.2 Preference differences of impressionistic oil paintings in six common interior design styles

The PAD emotional state model was used to evaluate emotional responses to Impressionistic oil paintings across six interior design styles by age and sex groups (Tables 5, 6).

**Table 5:** PAD emotion testing results and emotion categories for six interior design styles by age and sex.

Interior style	Yong				Yong-man				Yong-women				elderly			
	Py	Ay	Dy	emotions type	Pm	Am	Dm	emotions type	Pw	Aw	Dw	emotions type	Pe	Ae	De	emotions type
Nordic	0.5524	0.2119	0.1214	Joyful	0.3750	0.1083	#######	Dependent	0.7889	0.3500	######	Joyful	0.5833	0.4583	0.1417	Joyful
Franch	0.3810	-0.1595	#######	Mild	0.0542	#######	#######	Mild	0.8167	#######	######	Relaxed	0.3000	0.1583	0.1917	Joyful
American	0.6929	0.2429	0.0881	Joyful	0.4542	0.1000	#######	Dependent	1.0111	0.4333	######	Joyful	0.1750	#######	0.1917	Relaxed
pastoralism	-0.4595	0.2667	0.1214	Digust	#######	0.2708	0.0958	Digust	#######	0.2611	######	Digust	0.6083	0.4333	0.2917	Joyful
Modern	0.4190	0.0262	#######	Dependent	0.1750	0.0042	#######	Dependent	0.7444	0.0556	######	Dependent	0.3333	0.1333	0.1250	Joyful
New Chinese	0.1881	-0.0452	#######	Mild	0.0500	0.0167	#######	Dependent	0.3722	#######	######	Relaxed	#######	#######	#######	Boring

The “#” symbols in the table represent specific numerical values that are not displayed.

**Table 6:** Minimum PAD emotion proximity values for six interior design styles by age and sex.

**Volunteer**	**Interior** **style**	**Positive emotion**				**Negative emotion**			
		**Joyful**	**Relaxed**	**Surprise**	**Dependent**	**Boring**	**Fear**	**Contempt**	**Digust**
Yong	Nordic	2.7569	1.9480	1.9019	1.9066	2.0574	1.6833	2.3165	2.4228
	Franch	3.1260	2.2304	2.3165	**1.5624**	**1.6211**	1.5254	2.2892	2.3668
	American	**2.6503**	**1.8827**	**1.7958**	1.9129	2.1413	1.7946	2.4577	2.5647
	pastoralism	3.6064	2.9491	2.6160	2.1084	1.7971	**0.8989**	**1.4373**	**1.4545**
	Modern	3.0242	2.2907	2.1474	1.6404	1.7674	1.5084	2.2953	2.3686
	New Chinese	3.2060	2.3581	2.3408	1.6718	1.6312	1.3478	2.0772	2.1469
Yong-man	Nordic	3.0023	**2.1514**	2.1048	1.7277	1.8281	1.4753	2.2229	2.2994
	Franch	3.4367	2.5285	2.5708	**1.5131**	**1.3836**	1.2439	2.0686	2.1111
	American	**2.9488**	2.1530	**2.0631**	1.7138	1.8570	1.5431	2.2993	2.3791
	pastoralism	3.4839	2.7840	2.4935	2.0359	1.7989	**0.9673**	**1.5694**	**1.5997**
	Modern	3.2441	2.5037	2.3248	1.6095	1.6141	1.2797	2.1102	2.1594
	New Chinese	3.3141	2.4950	2.3936	1.6864	1.6015	1.1952	1.9733	2.0218
Yong-woman	Nordic	2.4303	1.6882	1.6504	2.1663	2.3689	1.9748	2.4745	2.6149
	Franch	2.7223	1.8525	2.0167	1.7321	1.9843	1.9297	2.6230	2.7360
	American	**2.2536**	**1.5238**	**1.4605**	2.2148	2.5268	2.1502	2.7071	2.8433
	pastoralism	3.7324	3.1371	2.7455	2.1965	1.8114	**0.8576**	**1.2998**	**1.3028**
	Modern	2.7385	2.0189	1.9352	1.7367	2.0016	1.8213	2.5588	2.6579
	New Chinese	3.0685	2.1771	2.2854	**1.6753**	**1.6928**	1.5528	2.2254	2.3198
Elderly	Nordic	2.6421	2.0189	1.6926	2.0678	2.2631	1.7221	2.3389	2.4419
	Franch	2.9522	2.2222	2.1035	1.9340	1.9330	1.5046	2.0607	2.1673
	American	3.2097	2.2267	2.4763	1.7841	1.6162	1.4959	2.0157	2.1260
	pastoralism	**2.5591**	**1.8072**	**1.6943**	2.1754	2.3260	1.8140	2.3091	2.4381
	Modern	2.9620	2.3448	2.1018	1.8626	1.8948	1.5006	2.1205	2.2179
	New Chinese	3.6725	2.7621	2.7876	**1.5941**	**1.2648**	**1.0600**	**1.8608**	**1.8796**

The bold values indicate the smallest PAD emotion proximity value among these several types of samples style.

For the younger group, Pleasure (P) scores were highest in American (P ≈ 0.69), Nordic (P ≈ 0.55), and Modern (P ≈ 0.42) interiors, indicating generally joyful or dependent emotions. These values were clearly higher than those for French (P ≈ 0.38) and New Chinese (P ≈ 0.19) styles, while Pastoralism showed a negative *p* value (P ≈ −0.46), reflecting a tendency toward dislike. Participants exhibited their highest Pleasure ratings for the American style and their lowest for Pastoralism, and the comparison between these two styles resulted in the largest effect size among all conditions (SMD = 1.00). This pattern suggests that the freedom, openness, and comfort of American interiors, as well as the minimalist and open spatial language of Nordic and Modern styles, better align with the aesthetic preferences of younger individuals (Coburn et al., 2020). The minimum proximity values in Table 6 further show that American interiors produced the most positive emotional matching in this group, whereas Pastoralism and, to a lesser degree, French interiors elicited more negative emotional tendencies, which is consistent with the values and aesthetic choices commonly associated with Generation Z (Peng, 2023).

In contrast, the elderly group showed a reversed pattern. Pastoralism yielded the highest P score (P ≈ 0.61), which was notably higher than that for American (P ≈ 0.18) and Modern (P ≈ 0.33) styles. Meanwhile, elderly participants expressed far more positive Pleasure responses than younger participants for Pastoralism, with a large effect (SMD = 1.00). This reflects the elderly participants’ preference for design environments incorporating natural elements and cultural depth (Korpela et al., 2010). By comparison, New Chinese interiors combined with Impressionistic paintings produced a negative p value (P ≈ −0.23), indicating boredom or rejection. Although elderly participants generally prefer culturally rich designs, the aesthetic and cultural mismatch between Western Impressionism and New Chinese interiors appears to create visual dissonance and emotional distance in this group, which tends to favor stability and familiarity (Lawton, 1973). PAD proximity values corroborate this trend: Pastoral interiors show the smallest distance to positive emotional norms, whereas New Chinese interiors are closest to negative emotion norms for elderly participants.

The Arousal (A) and Dominance (D) dimensions further support these tendencies. Among younger participants, Nordic interiors produced a higher arousal score (A ≈ 0.21) than Modern interiors (A ≈ 0.03), and a higher dominance score (D ≈ 0.12 vs. –0.07), suggesting that Nordic design language may enhance both emotional activation and perceived control in this group. For the elderly group, Pastoral interiors showed a clearly higher arousal level (A ≈ 0.43) than Modern interiors (A ≈ 0.13), and also the highest dominance score (D ≈ 0.29), indicating that familiar and warm environments significantly strengthen both emotional engagement and the psychological sense of control (Kaplan and Kaplan, 1989).

With regard to sex differences, female and male participants exhibited broadly similar preference patterns: both groups reported their highest *p* values for American interiors (Pw ≈ 1.01, Pm ≈ 0.45) and their lowest p values for Pastoralism (Pw ≈ −0.61, Pm ≈ −0.31). However, female participants generally showed stronger emotional reactions across styles (Table 5), which is in line with previous findings on gender differences in aesthetic sensitivity (McManus and Furnham, 2006). For instance, Female participants also exhibited stronger aversion to Pastoralism than males (SMD = −0.32). From the perspective of positive–negative emotional proximity, Pastoral interiors displayed the largest gap between positive and negative emotion scores, suggesting a strong polarizing effect and making this style particularly relevant for discussions of emotional regulation in environmental design (Kaplan and Kaplan, 1989).

Overall, these results indicate that interior design style meaningfully shapes emotional responses to Impressionistic oil paintings, with Modern, American, and Nordic interiors being more suitable for young individuals, while Pastoral and other traditional styles more effectively meet the emotional needs of elderly participants.

#### 3.1.3 Preference classification analysis of oil painting styles and interior design styles

A preference classification analysis was conducted for the eight oil painting styles and Impressionistic paintings across six interior design styles based on the Pleasure (P) values and emotional state categories of the younger group in Tables 3 and 5. The results are presented in Table 7. For the oil painting styles, the *p* values were classified into four nodes: −1.25, −0.25, 0.25, and 1.25. The Romanticism, Impressionistic, and Post-Impressionistic styles were categorized as “like.” Renaissance, Baroque, Modernism, and Abstractionism were considered “neutral.” Contemporary art was classified as “dislike.” For interior design styles, the p values were classified into four nodes: −1.5, −0.2, 0.5, and 1.5. The Nordic and American styles were classified as “like.” The French, Modern, and New Chinese styles were considered as “neutral.” The Pastoralism style was classified as “dislike.” These classification results might serve as the basis for subsequent EEG experiments to induce different emotional experiences in volunteers.

**Table 7:** Classification of Pleasure values for eight oil painting styles and six interior design styles.

Ground	Oil painting style	Pleasure (P)	Interior style	Pleasure (P)
Pleased	Romanticism, Impressionistic, Post-impressionistic	[0.25 ~ 1.25]	Nordic, American	[0.5 ~ 1.5]
General	Renaissance, Baroque, modernism, abstractionism	[−0.25 ~ 0.25]	Franch, Modern, New Chinese	[−0.2 ~ 0.5]
Unpleased	Contemporary art	[−1.25 ~ −0.25]	Pastoralism	[−1.5 ~ −0.2]

### 3.2 ERP (event related potentials) preference analysis

#### 3.2.1 Overall ERP data preference analysis

**Figure 4:**
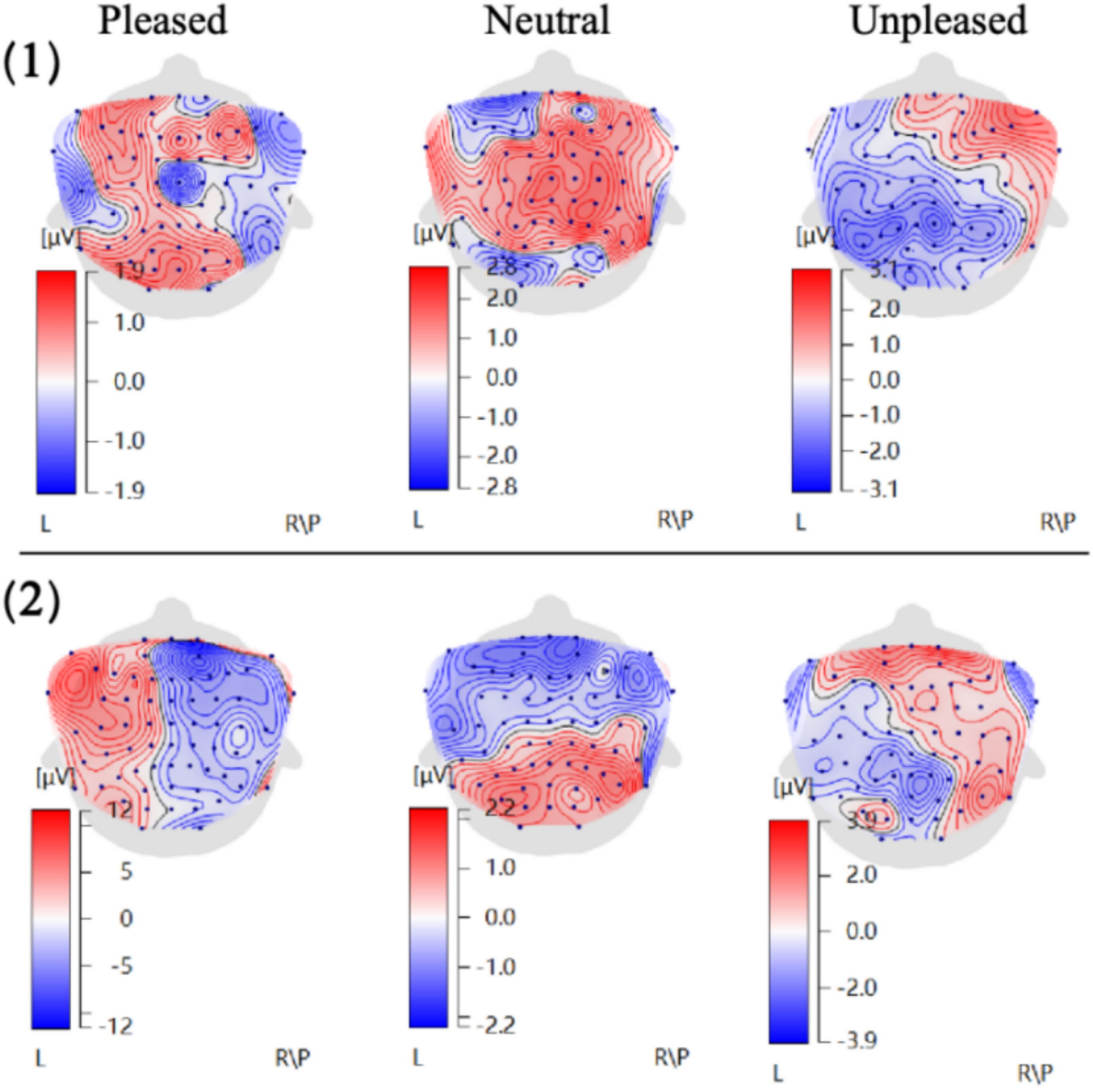
ERP topographic maps induced by different oil painting and interior design styles. (1) Average topographic maps for eight oil painting styles. (2) Average topographic maps for six interior design styles.

**Figure 5:**
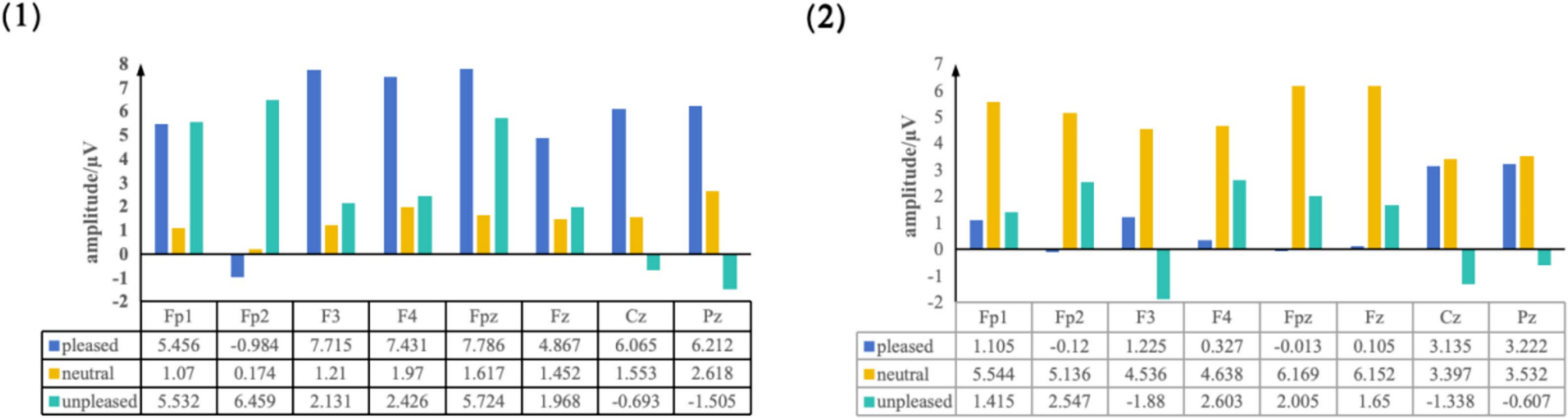
Average maximum ERP amplitudes of different electrodes. (1) Average maximum ERP amplitudes induced by preferences among eight oil painting styles. (2) Average maximum ERP amplitudes induced by preferences among six interior design styles.

Three types of ERP topographic maps corresponding to “like,” “neutral,” and “dislike” emotional states were obtained by averaging EEG data from 35 participants, as shown in Figure 4. The map indicated broadly that, regardless of different oil painting styles or interior design styles, the emotion-induced brain activity patterns were similar. Specifically, topographic maps for the “like” emotion showed stronger activation in the left hemisphere, whereas those for the “dislike” emotion showed stronger activation in the right hemisphere. When the left and right hemisphere activations were equivalent, a neutral emotional state was observed, which was consistent with the “valence hypothesis” (Briesemeister et al., 2013). Moreover, the maximum ERP amplitudes were extracted from eight electrodes (Fp1, Fpz, Fp2, F3, F4, Fz, Cz, and Pz) between 250 and 600 ms, which corresponds to the time window of late positive potential (LPP) commonly associated with affective visual processing. The average values are shown in Figure 5.

The maximum amplitude data from these eight electrode sites indicated that the left frontal lobe electrodes (FP1 and F3) had greater amplitude values than the right frontal lobe counterparts (FP2 and F4) in both datasets. This indicated a tendency toward higher left hemisphere activation in the liked oil painting styles. In contrast, the disliked paintings elicited higher right hemisphere amplitudes, which was consistent with interpretations of emotional lateralization in the left hemisphere (Coan and Allen, 2004) and induction of stronger negative emotions in the right hemisphere (Schmidt, 1999). Additionally, central (Cz) and parietal (Pz) regions showed higher amplitudes for the liked samples, indicating higher emotional arousal levels (Harmon-Jones, 2003). However, the maximum ERP amplitudes in neutral emotional states of various painting styles were lower than those in different interior environments. This suggested that the complex elements in interior environments required participants to process more complex stimuli, thus eliciting higher levels of emotional arousal.

#### 3.2.2 Preference difference analysis of eight common oil painting styles in modern interior environments

**Figure 6:**
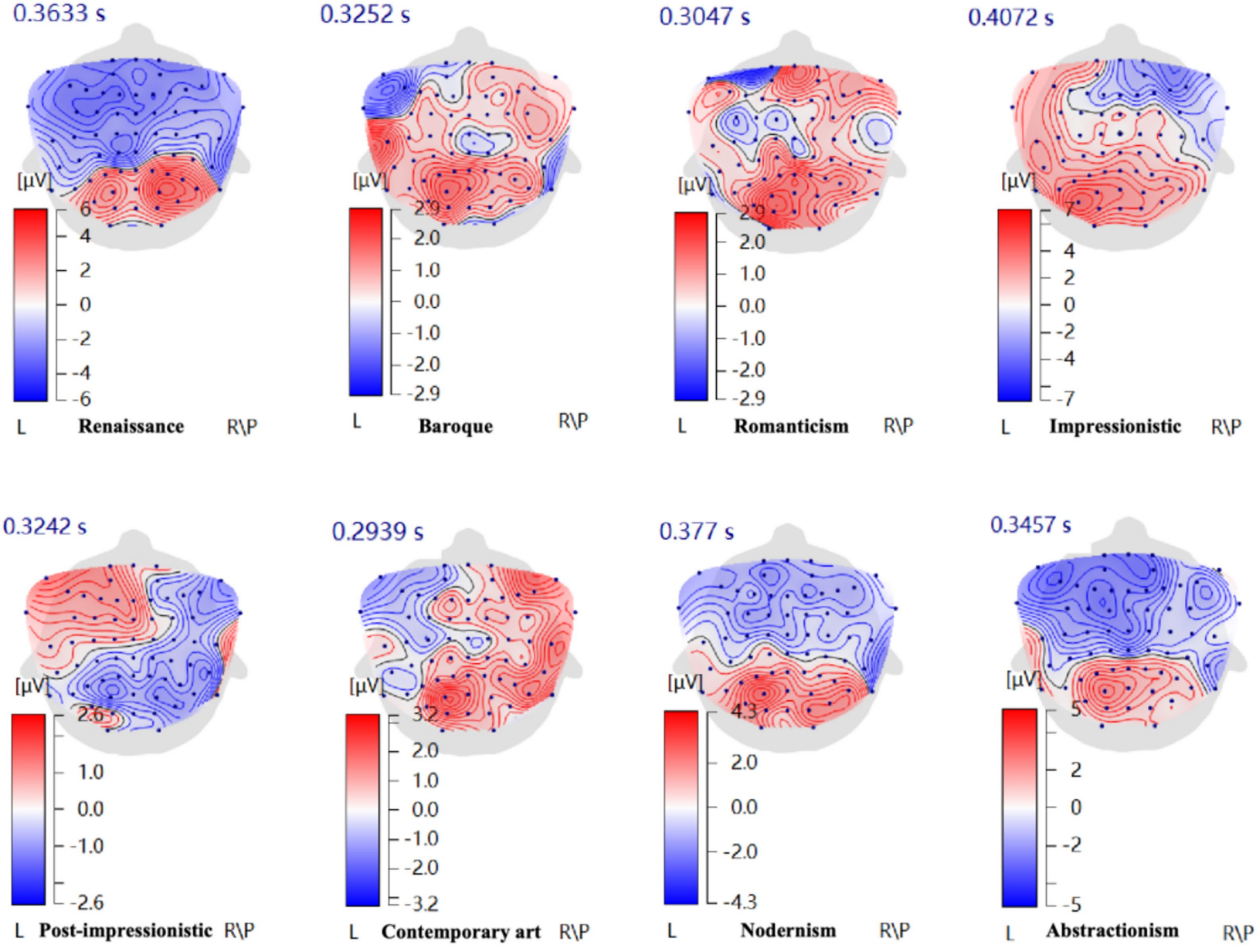
ERP topographic maps induced by preference for eight oil painting styles.

Based on the “valence hypothesis,” this study analyzed ERP topographic maps elicited by eight oil painting styles (Figure 6). The results showed that Romanticism, Impressionistic, and Post-Impressionistic paintings primarily induced positive emotions in the left hemisphere, indicating a higher preference among participants for these styles. In contrast, Contemporary art exhibited strong right hemisphere activation, suggesting that the participants rejected this style. The ERP maps of Renaissance, Baroque, Modernism, and Abstractionism displayed balanced activation between hemispheres, reflecting a neutral stance. This was consistent with the results of PAD preference analysis.

**Figure 7:**
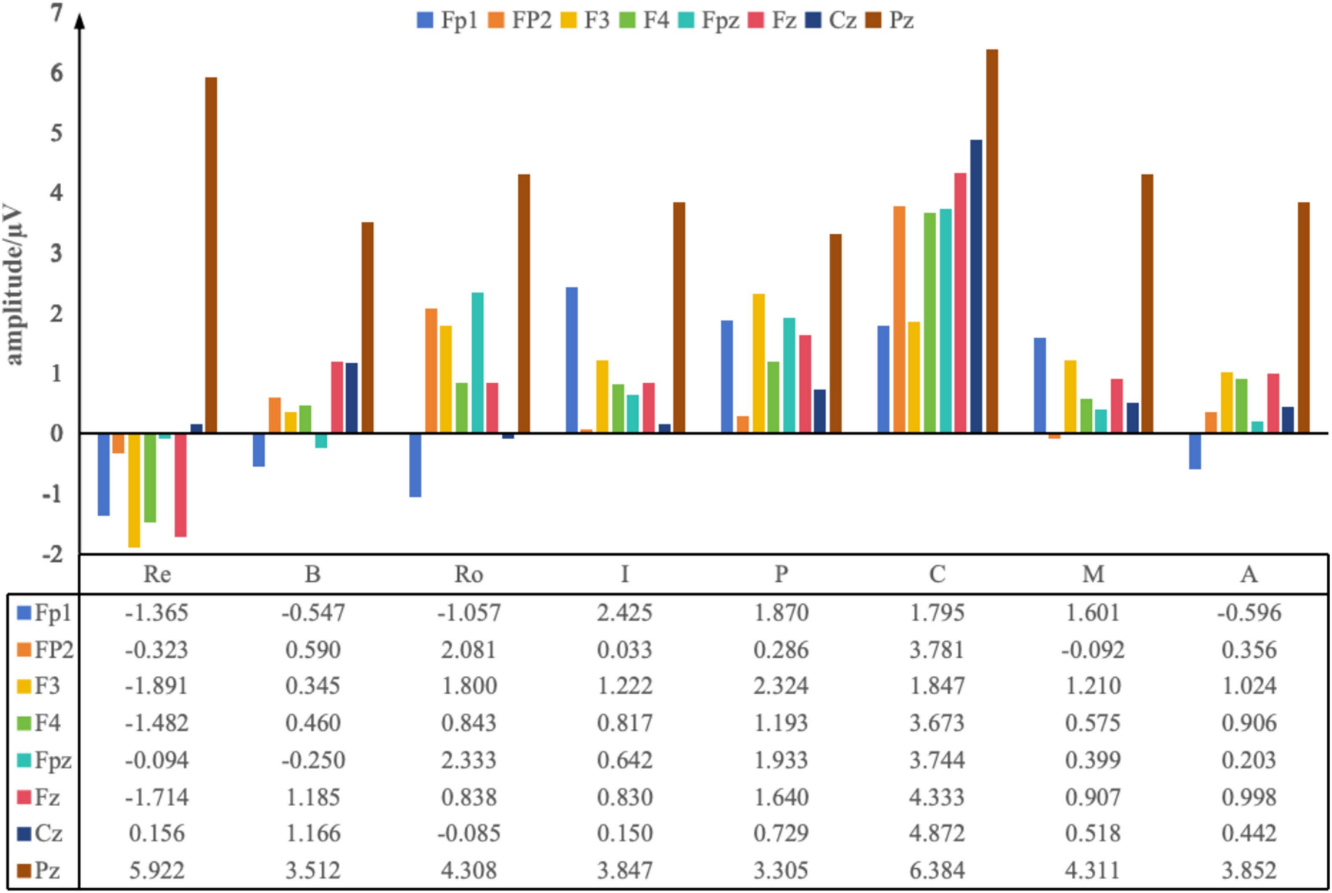
Average maximum ERP amplitudes induced by eight oil painting styles. Re, Renaissance; B, Baroque; Ro, Romanticism; I, Impressionistic; P, Post-Impressionistic; C, Contemporary art; M, Modernism; A, Abstractionism.

Further examination of the maximum ERP amplitudes (Figure 7) revealed neural activation patterns that help explain the hemispheric preference tendencies described above. The Renaissance style showed negative-going frontal waveforms (Fp1: −1.365 μV; F3: −1.891 μV; Fp2: −0.323 μV) accompanied by the highest parietal activation (Pz: 5.922 μV). This combination—frontal suppression with strong parietal engagement—reflects deep perceptual and semantic processing rather than a clear approach tendency, aligning with its neutral hemispheric pattern (Davidson, 1992). Baroque elicited high central amplitudes (Fz: 1.185 μV; Cz: 1.166 μV), indicating strong attentional engagement, but without clear left–right dominance, again consistent with its neutral preference tendency (Harmon-Jones and Gable, 2018). Romanticism exhibited strong frontal amplitudes in both hemispheres (Fp2: 2.081 μV; F3: 1.800 μV), but with left-frontal predominance (F3 > F4), indicating an approach-oriented emotional tendency. The relatively large amplitudes suggest heightened emotional engagement, supporting the preference indicated by its topographic maps (Vartanian and Skov, 2014). The Impressionistic style showed pronounced left-frontal activation (Fp1: 2.425 μV) and moderate parietal activity (Pz: 3.847 μV). The leftward asymmetry indicates preference, while the amplitude magnitude reflects smooth perceptual processing and moderate emotional involvement, matching its high preference in the PAD and hemispheric analysis (Coan and Allen, 2004). Post-Impressionism elicited high parietal amplitudes (Pz: 3.305 μV), indicating increased perceptual load associated with its strong color contrasts (Chatterjee, 2004). The absence of strong frontal asymmetry corresponds to its moderate preference level in the first analysis. Modern and Contemporary art styles both generated strong central/parietal amplitudes (Modern Cz: 4.311 μV; Contemporary Pz: 6.384 μV), suggesting high cognitive demands. Importantly, Contemporary art showed dominant right-frontal activation (Fp2: 3.781 μV; F4: 3.673 μV), corresponding to the withdrawal-related, low-preference tendency observed in the topographic maps. The large amplitudes further indicate high processing difficulty and ambiguity, which may contribute to this decreased preference (Schmidt, 1999). Abstractionism produced a negative-going frontal waveform (Fp1: −0.596 μV) with high parietal activation (Pz: 3.852 μV). The relatively right-weighted frontal pattern reflects a slight withdrawal tendency, while the high parietal amplitudes indicate substantial cognitive demand, aligning with its neutral-to-lower preference in the first analysis (Yang and Lee, 2020).

Overall, by combining frontal hemispheric asymmetry (indicating the direction of preference) with amplitude magnitude and waveform characteristics (indicating the intensity and processing load associated with each style), the ERP findings provide a coherent explanation for the emotional and preference tendencies revealed across the eight painting styles.

#### 3.2.3 Preference difference analysis of impressionistic style in six common interior design styles

**Figure 8:**
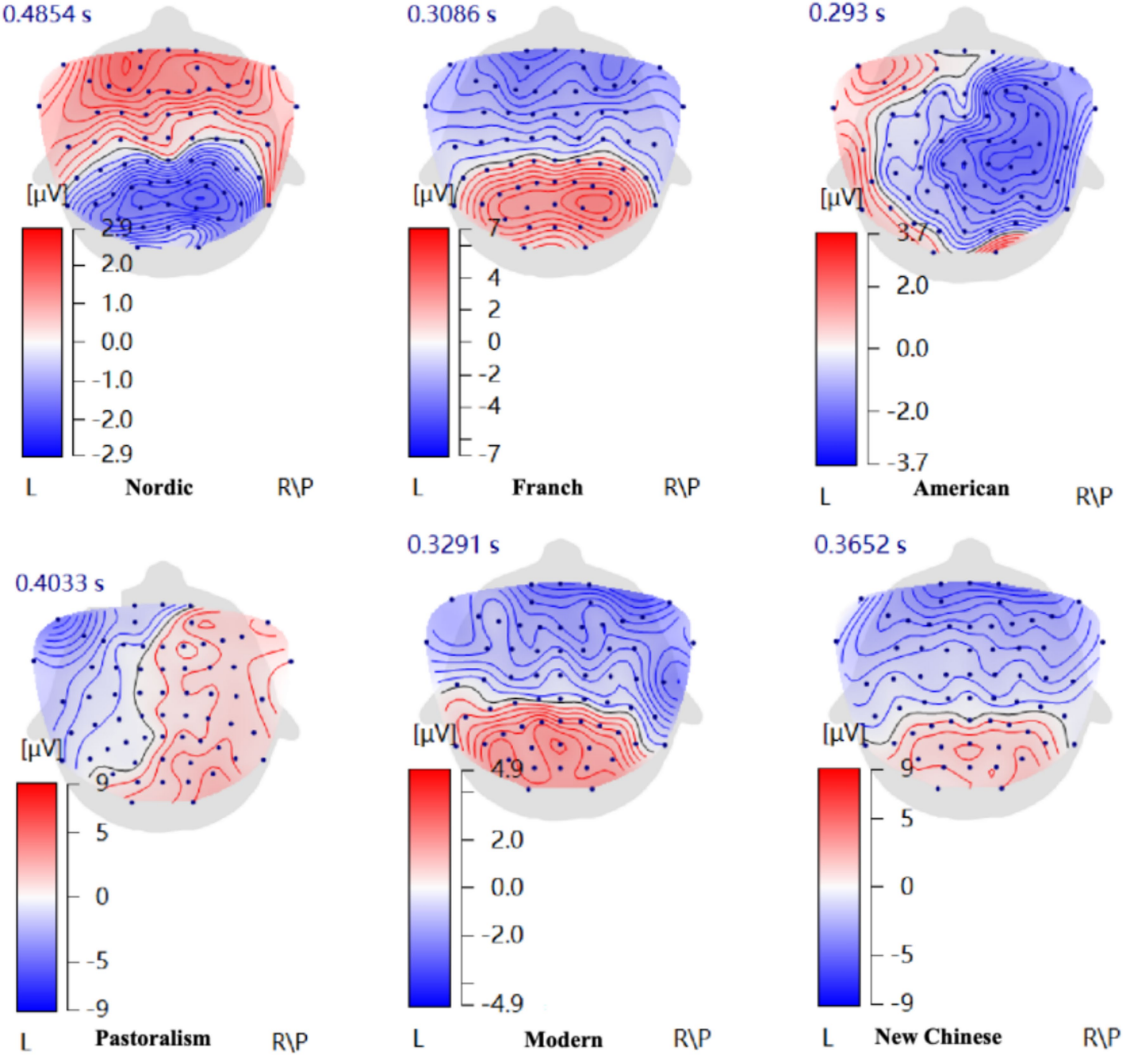
ERP topographic maps induced by impressionistic paintings in six interior design styles.

Following the aforementioned “valence hypothesis,” ERP topographic maps induced by Impressionistic paintings in six interior environments (Figure 8) showed that Impressionistic paintings in the American-style interior environment induced positive emotions in the left hemisphere, indicating the preference of participants for Impressionistic paintings in this interior style. In contrast, Impressionistic paintings in the Pastoralism-style environment induced strong right hemisphere activation, suggesting that participants disliked the combination of Impressionistic paintings with this interior style. The ERP topographic maps for Nordic, French, Modern, and New Chinese styles exhibited balanced activation between the left and right hemispheres, indicating neutral emotions toward these four styles. These findings were consistent with the PAD preference analysis presented earlier.

**Figure 9:**
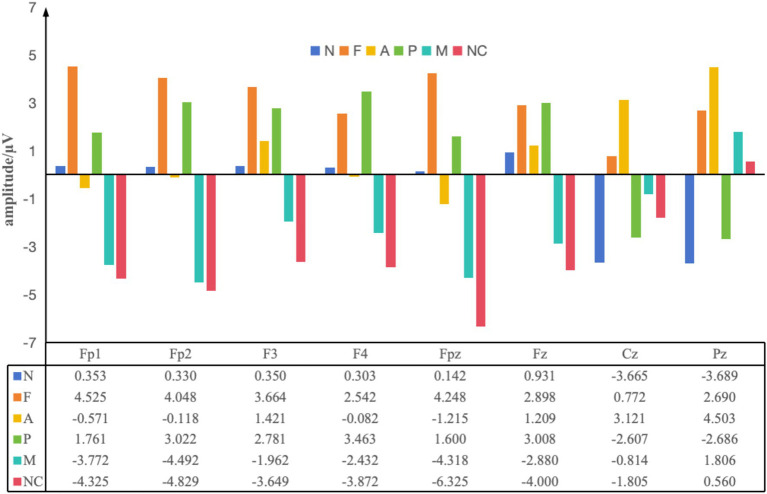
Average maximum ERP amplitudes induced by impressionistic paintings in six interior design styles: N, Nordic; F, French; A, American; P, Pastoralism; M, Modern; NC, New Chinese.

Further analysis of the maximum ERP amplitudes generated by Impressionistic paintings in each of the six interior design styles (Figure 9) provides additional insight into the intensity and processing characteristics underlying these preference tendencies. The Nordic style produced moderate frontal amplitudes (Fp1: 0.353 μV; Fp2: 0.330 μV) and a relatively high parietal response (Pz: 3.689 μV), indicating smooth perceptual processing and a low-arousal, pleasant experience, consistent with the PAD results (Davidson, 1992). The American style elicited a large parietal response (Pz: 4.503 μV) but comparatively low frontal amplitudes (Fp1: −0.571 μV; Fp2: −0.118 μV). This pattern—high perceptual engagement combined with left-frontal hemispheric preference in the earlier analysis—suggests that the style’s openness and visual coherence may enhance the sense of environmental comfort and control (Harmon-Jones and Gable, 2018). Pastoralism evoked relatively high frontal amplitudes (Fp1: 1.761 μV; Fp2: 3.022 μV) but negative-going waveforms in the parietal region (Pz: −2.686 μV). Rather than indicating “negative emotion,” this pattern reflects higher frontal effort coupled with reduced perceptual processing, which corresponds to the withdrawal tendency observed in the hemispheric maps and the low Pleasure and Dominance values in the PAD model (Vartanian and Skov, 2014). The French style induced stronger activation in both the left frontal (Fp1: 4.525 μV) and parietal regions (Pz: 2.690 μV), suggesting elevated emotional engagement and aesthetic appreciation of its refined design elements. However, the lower Dominance scores in the PAD model indicate that participants experienced this style more as passive admiration than active approach (Coan and Allen, 2004). For the New Chinese style, negative-going frontal amplitudes (Fp1: −4.325 μV; Fp2: −4.829 μV) paired with mildly positive-going parietal activation (Pz: 0.560 μV) reflect increased cognitive processing demands due to its dense cultural symbolism, without necessarily producing a clear approach or withdrawal tendency (Chatterjee, 2004). Similarly, the Modern style showed negative-going frontal amplitudes (Fp1: −3.772 μV; Fp2: −4.492 μV) with modest parietal activation (Pz: 1.806 μV), suggesting that its cool and simplified aesthetic prompted limited emotional engagement but maintained perceptual clarity (Yang and Lee, 2020).

Together, these results demonstrate how interior design styles shape participant preference by jointly influencing motivational direction (indicated by frontal hemispheric asymmetry) and processing intensity (indicated by ERP amplitude patterns). Nordic and American styles elicited stronger approach-related tendencies and moderate-to-high engagement, aligning with their overall higher preference. Pastoralism showed withdrawal-related tendencies combined with less efficient perceptual processing, resulting in lower preference. French, New Chinese, and Modern styles produced more nuanced combinations of hemispheric and amplitude patterns, consistent with their neutral preference levels in the PAD analysis.

## 4 Conclusion

This study explored emotional responses and preference differences for various decorative oil painting styles in multiple interior environments using the PAD emotional state model and EEG. The findings suggested that the combination of different painting styles and interior environments significantly impacted the emotional experiences and aesthetic preferences of individuals. Specifically, Impressionistic, Post-Impressionistic, and Romanticism painting styles were tended to evoke more positive emotions in modern and Nordic interior environments and were favored by younger and female participants. In contrast, Contemporary art was less preferred, especially in Pastoralism and New Chinese interior environments, exhibiting strong negative emotions.

Moreover, the results from the PAD emotion scales and EEG experiments were highly consistent, showing general correspondence with the valence hypothesis. This indicated positive emotions were more likely to activate the left frontal lobe, whereas negative emotions were more likely to activate the right frontal lobe. The EEG data showed that painting styles with high Pleasure scores (e.g., Impressionistic style) induced stronger positive-going amplitudes in the frontal regions, whereas painting styles with low Pleasure scores (e.g., Contemporary art) showed higher negative-going amplitudes. Furthermore, different age and sex groups exhibited variations in oil painting style and interior environment preferences. The elderly participants preferred comfortable and culturally rich styles, whereas the younger participants favored novelty and visual impact.

These findings hold significant theoretical and practical implications for the art and interior design fields. Individual emotional experiences can be more precisely evaluated by combining PAD emotional measurement and EEG neurophysiological data, thereby providing data support for interior designers, art curators, and environmental psychology researchers. Also, the results provide a scientific basis for optimizing visual experiences in environmental design and art applications. Future studies should further explore more types of artworks and other physiological measurement techniques to fully reveal the complex relationship between visual art and emotional experience.

## Data Availability

The original contributions presented in the study are included in the article/supplementary material, further inquiries can be directed to the corresponding author.
